# Ring Trial on Quantitative Assessment of Bile Acids Reveals a Method- and Analyte-Specific Accuracy and Reproducibility

**DOI:** 10.3390/metabo12070583

**Published:** 2022-06-23

**Authors:** Sven-Bastiaan Haange, Andreas Till, Per-Olof Bergh, Günter Fauler, Michael Gigl, Anita Löfgren-Sandblom, Frank G. Schaap, Thomas Clavel, Christian Trautwein, Wiebke Fenske, Karin Kleigrewe, Hanns-Ulrich Marschall, Steven W. M. Olde Damink, Tarek Moustafa, Martin von Bergen, Ulrike Rolle-Kampczyk

**Affiliations:** 1 Department of Molecular Systems Biology, Helmholtz Centre for Environmental Research—UFZ, 04318 Leipzig, Germany; sven.haange@ufz.de (S.-B.H.); martin.vonbergen@ufz.de (M.v.B.); 2 Department of Internal Medicine I, Division of Endocrinology, Diabetes, and Metabolism, University Medical Center Bonn, 53127 Bonn, Germany; a.till@uni-bonn.de (A.T.); wiebke.fenske@ukbonn.de (W.F.); 3 Wallenberg Laboratory, Department of Molecular and Clinical Medicine, University of Gothenburg, 413 45 Gothenburg, Sweden; per-olof.bergh@wlab.gu.se (P.-O.B.); hanns-ulrich.marschall@gu.se (H.-U.M.); 4 Clinical Institute of Medical and Chemical Laboratory Diagnostics, Medical University of Graz, 8036 Graz, Austria; guenter.fauler@medunigraz.at; 5 Bavarian Center for Biomolecular Mass Spectrometry, TUM School of Life Sciences, Technical University of Munich, 85354 Freising, Germany; michael.gigl@tum.de (M.G.); karin.kleigrewe@tum.de (K.K.); 6 Department of Laboratory Medicine, Karolinska Institute, 171 77 Stockholm, Sweden; anita.lovgren.sandblom@ki.se; 7 Department of Surgery, NUTRIM School of Nutrition and Translational Research in Metabolism, Maastricht University, 6229 ER Maastricht, The Netherlands; frank.schaap@maastrichtuniversity.nl (F.G.S.); steven.oldedamink@maastrichtuniversity.nl (S.W.M.O.D.); 8 Department of General, Visceral and Transplant Surgery, University Hospital Aachen, 52074 Aachen, Germany; 9 Functional Microbiome Research Group, Institute of Medical Microbiology, University Hospital of RWTH Aachen, 52074 Aachen, Germany; tclavel@ukaachen.de; 10 Medizinische Klinik III (Gastroenterology, Metabolic Disease and Intensive Care), University Clinic RWTH, 52074 Aachen, Germany; ctrautwein@ukaachen.de; 11 Division of Gastroenterology and Hepatology, Medical University of Graz, 8036 Graz, Austria; tarek.moustafa@medunigraz.at

**Keywords:** bile acids, ring trial, LC-MS/MS, absolute quantification, human serum, murine serum

## Abstract

Bile acids are a key mediator of the molecular microbiome-host interaction, and various mass spectrometry-based assays have been developed in the recent decade to quantify a wide range of bile acids. We compare existing methodologies to harmonize them. Methodology for absolute quantification of bile acids from six laboratories in Europe were compared for the quantification of the primary bile acids cholic acid (CA) and chenodeoxycholic acid (CDCA) and conjugated products glycocholic acid (GCA) and taurocholic acid (TCA). For the bacterially modified secondary bile acids, the quantification of deoxycholic acid (DCA) and lithocholic acid (LCA) was compared. For the murine bile acids, we used the primary muricholic acids (α-MCA and, β-MCA) and the intestinally produced secondary bile acid muricholic (ω-MCA). The standards were spiked into methanol:water (1:1) mix as well as in human and murine serum at either low concentration range (150–3000 nM) or high concentration range (1500–40,000 nM). The precision was better for higher concentrations. Measurements for the hydrophobic unconjugated bile acids LCA and ω-MCA were the most challenging. The quality assessments were generally very similar, and the comprehensive analyses demonstrated that data from chosen locations can be used for comparisons between studies.

## 1. Introduction

One of the most important mediators of crosstalk between the gut and the liver is bile acids (BAs). They are produced in the liver and secreted into the bile ducts that end up in the duodenum. To maintain a higher solubility in the bile fluid the primary BAs cholic acid and chenodeoxycholic acids are conjugated to either glycine or taurine, yielding taurocholic acid, taurochenodeoxycholic acid, and glycocholic acid and glycochenodeoxycholic acid [1]. After they are secreted into the gut, they can be deconjugated by bacterial bile acid hydrolases [2,3,4] that are widespread among bacteria in the small and especially in the large intestine. Besides deconjugation, bacterial enzymes also modify a large extent of deconjugated bile acids further to deoxycholic acid and lithocholic acid [5,6]. The modification of BAs impacts the toxicity of bacterial species because more hydrophobic bile acids are more toxic for bacteria and this impacts the bacterial composition in the large intestine [7], but they are also less soluble in the aqueous phase in the colon.

Bile acids are not only involved in solubilizing dietary lipids [8] but are also regulating the lipid and glucose metabolism in the liver via the farnesoid X receptor (FXR) [9]. The bile acids are part of a regulatory feedback loop by differential binding to the FXR, resulting in decreased synthesis of bile acids at high concentrations of bile acids [10]. These bile acid-specific feedback loops in combination with the modifying capacity of the microbiome result in stable maintenance of systemic bile acid levels. This demonstrates that the gut–liver axis is pivotal for many diseases associated with the liver like NAFLD and liver cancer [1,11,12].

Changes in the systemic bile acid profile and quantities are linked to many different health outcomes like the early microbiome development [13], NAFLD [14], liver cancer [15], and the change of the intestinal topology after bariatric surgery [16].

The qualitative assessment and relative quantification of BAs is useful for smaller case studies because it allows for identifying relevant specific bile acids. However, for further insights into the mechanisms like the feedback regulation and for modeling the overall bile acid metabolism, absolute quantification is required. Furthermore, only absolute quantification allows comparing the bile acid levels across several studies, which is of specific interest in the case of large prospective and retrospective cohort studies [17].

Nearly all quantification methods are mass spectrometry-based assays, most often in conjunction with a separation by HPLC prior to mass spectrometry-based detection. The great majority of applied mass spectrometers are employing a triple quadrupole mass analyzer using the multiple reaction monitoring modes (MRM) [18,19] but recently also orbitrap-based mass spectrometers are used for detecting bile acids [20]. The latter type of instrument has the advantage of a higher mass resolution [21] and consequently, it can be used for the targeted detection of a wide range of metabolites. In contrast, two disadvantages that high-resolution mass spectrometers have is that they lack both the high sensitivity and large dynamic linear range of triple quadrupole-based mass spectrometers, and therefore triple quadrupole mass spectrometers are able to accurately quantify compounds in a larger concentration range. One MRM transition is normally used as a quantifier and one MRM transition is used as a qualifier. 

The detection of metabolites can be affected by matrix effects, which might hamper especially the recovery of metabolites in the medium. This effect can be analyzed by spiking the analyte of interest into a matrix consisting of a solution that solubilizes bile acids well, such as methanol. The recovery with and without a complex matrix can then be determined. Thus, it would be ideal if a biological matrix would be available without bile acids, which is not the case. Therefore, the second-best approach is to spike in a known concentration of bile acid into the solution medium for determining the recovery and into pooled serum samples for determining the baseline.

The goal of this study was to examine six existing methods for absolute quantification of a wider spectrum of bile acids to conclude the general characteristics of bile acid detection as well as the strengths and shortcomings of the methods used. It will also clarify to what extent the outcomes of the various approaches may be compared, as well as the next steps toward method harmonization.

## 2. Results

This study aimed to assess the absolute quantitative methods of bile acids (BAs) in human and murine serum from 6 centers. To facilitate this, nine BAs were spiked together into human or murine serum at either low or high concentrations (see Table 1). The BAs were chosen to cover the main bile acid classes. Cholic acid (CA) and chenodeoxycholic acid (CDCA) were chosen as the primary human bile acids for analysis, while α-muricholic acid (α-MCA) and β-muricholic acid (β-MCA) as primary murine bile acids. Glycocholic acid (GCA) and taurocholic acid (TCA) were chosen as examples of glycine and taurine conjugated primary bile acids, respectively. For the class of secondary bile acids deoxycholic acid (DCA), lithocholic acid (LCA), and ω-muricholic acid (ω-MCA) were selected. Low and high concentrations represented common low or high BA values typically detected in serum samples. The differences between the two concentrations were a factor of ten for seven of the bile acids, while for the bile acids GCA and TCA the factor was one hundred (see Table 1). Center 3 method for bile acid quantification did not cover LCA or ω-MCA, while the Center 4 method did not cover α-MCA or ω-MCA. Mean BA concentrations are listed in the Supplementary Material in Supplemental Table S1.

**Figure 1:**
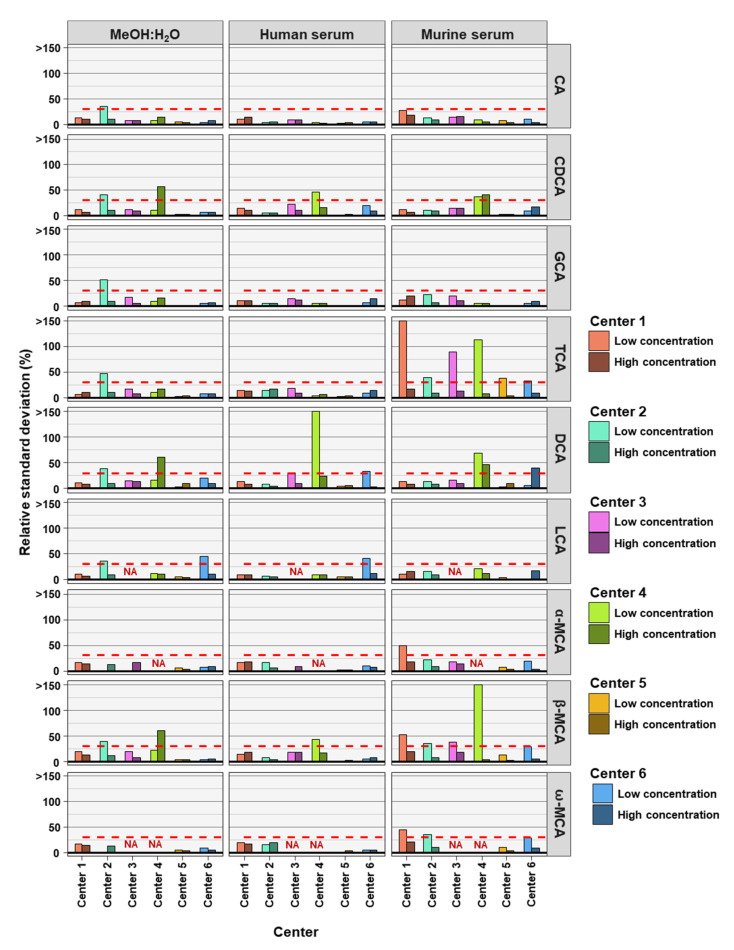
The precision of bile acid analyses of each assessing center. Relative standard deviations (n = 6) of the nine bile acids spiked into MeOH:H_2_0 solvent as well as human and murine serum at high and low concentration levels. The red dashed line represents a relative standard deviation of 30%. NA = not applicable, those BAs were not covered in the analysis of that center.

To determine the precision of the measurements the relative standard deviation (RSD) of each bile acid at high and low concentrations in the three analyzed matrices was determined. For precision, 30% was regarded as a maximum acceptable value for RSD (Figure 1). 

As frequently observed in analytics, lower concentrations are associated with larger deviations in measurements. Here we found that most measurements outside the precision threshold were from those with low concentrations spiked into the matrices. This holds true for all three matrices. DCA (8 times), TCA (8 times), and β-MCA (7 times) were the BAs with the highest number of analyses outside precision limits. CA (35 times out of 36 analyses), GCA (35 times out of 36 analyses) and LCA (27 times out of 30 analyses) measurements were predominantly within precision limits, for the three matrices, two concentrations, and six assessing centers. In total, of the 54 or 42 BA analyzed (nine or seven BAs, at two concentrations and in three matrices) center 1 reached acceptable precision in 50 out of 54 cases, center 2 in 43 out of 54 cases, center 3 in 37 out of 42 cases, center 4 in 30 out of 42 cases, center 5 in 53 out of 54 cases, and center 6 in 48 out of 54 cases (see Table 2). For the majority of centers, precision was reached most often for BAs in MeOH:H_2_O matrix.

**Figure 2:**
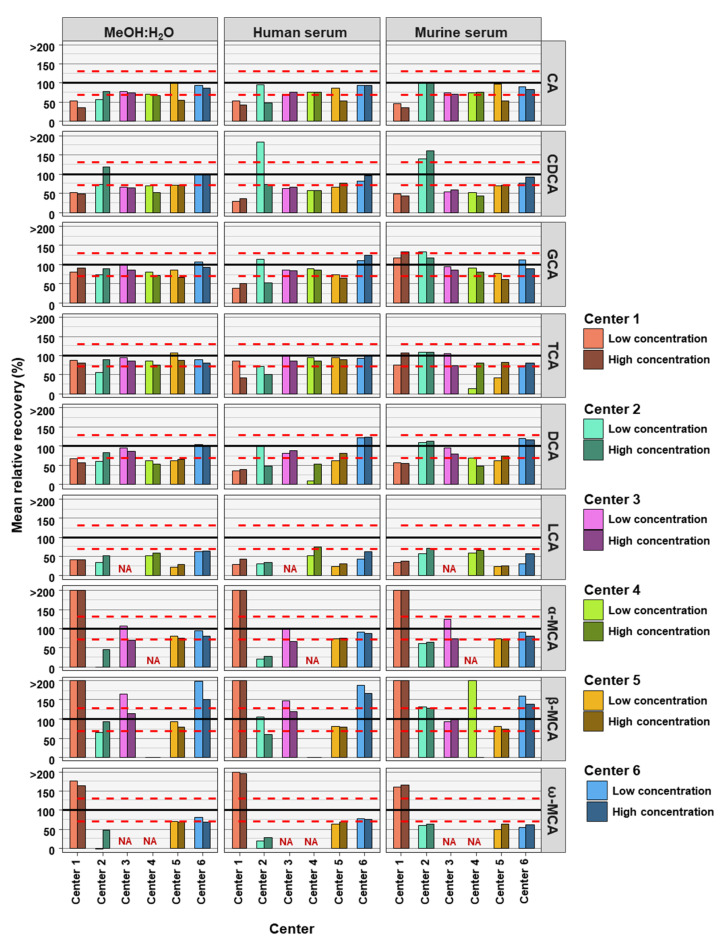
Accuracy of bile acid analyses of each assessing center. Mean relative recovery (n = 6) of the nine bile acids spiked into the MeOH:H_2_O solvent, as well as human and murine serum, at high and low concentration levels. The red dashed line represents relative recovery values of 70% (lower line) and 130% (higher line). NA = not applicable, those BAs are not covered by the specific center.

The accuracies of the BA analyses were determined by the relative recoveries. A relative recovery of between 70% and 130% was deemed to be acceptable (see Figure 2). Accuracy thresholds were reached in fewer cases than precision thresholds (Table 2 and Table 3). In total, for the 54 or 42 (centers 3 and 4) BAs analyzed (nine or seven BAs, at two concentrations and in three matrices) each assessing center performed as follows: center 1 was within acceptable accuracy in 8 out of 54 cases, center 2 in 20 out of 54 cases, center 3 in 32 out of 42 cases, center 4 in 17 out of 42 cases, center 5 in 27 out of 54 cases, and center 6 out in 39 of 54 cases (see Table 3). There were no great differences between matrix and concentration levels of BAs in the number of analyses within acceptable accuracy when considering all assessing centers. Human serum with BAs at low concentration had most BA analyses within relative recovery limits (viz. 25), while human serum with high concentrations of BAs had the least BA analysis within relative recovery limits (viz. 22). Summarizing BA analyses from all assessing centers at low and high concentrations in the three matrices, analyses of CA (24 times) and its conjugates GCA (28 times) and TCA (31 times) were most often within accuracy limits, while ω-MCA (4 times) and LCA (1 time) were the BAs reaching accuracy limits the fewest times.

**Figure 3:**
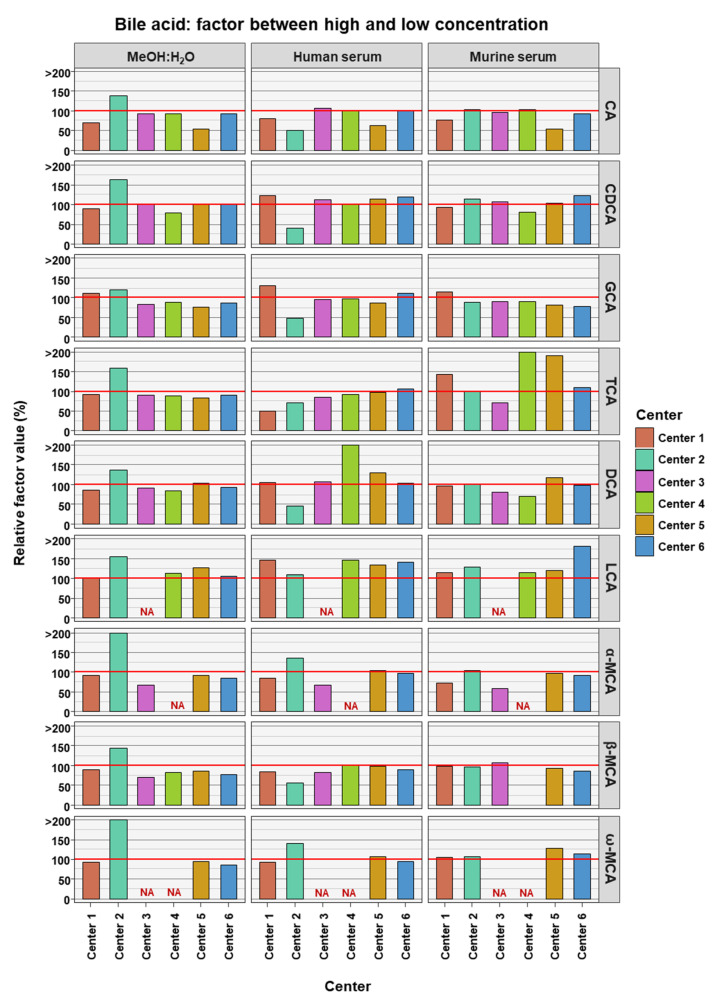
Relative factor values derived from the experimental factors, calculated from the mean bile acid concentrations measured in samples between the high and the low concentrations, and theoretical factors, calculated from the known spike-in concentrations. NA = not applicable, those BAs are not covered by the specific center.

To access if BA measurements in low and high concentrations are comparable to each other, the relative factor value was calculated between the factor reached in the experimental measurements and compared to the theoretical factor. The theoretical factor was 10 apart from TCA and GCA where it was 100. This was done for data from each assessing center, for each BA in each of the three matrices (see Figure 3). The experimental factor was generally relatively close to the theoretical values. LCA seemed to have experimental factors generally higher than the theoretical values in the two serum matrices and across all assessing centers (relative factor value: human serum 134.6% ± 12.9% SD; murine serum 131.4% ± 25.9% SD). For DCA in human serum (164.3% ± 148.5% SD) and TCA in murine serum (193% ± 162.1% SD), the experimental factors were far greater than the theoretical factor, though in these cases this was due to an outlier measurement in each case.

**Figure 4:**
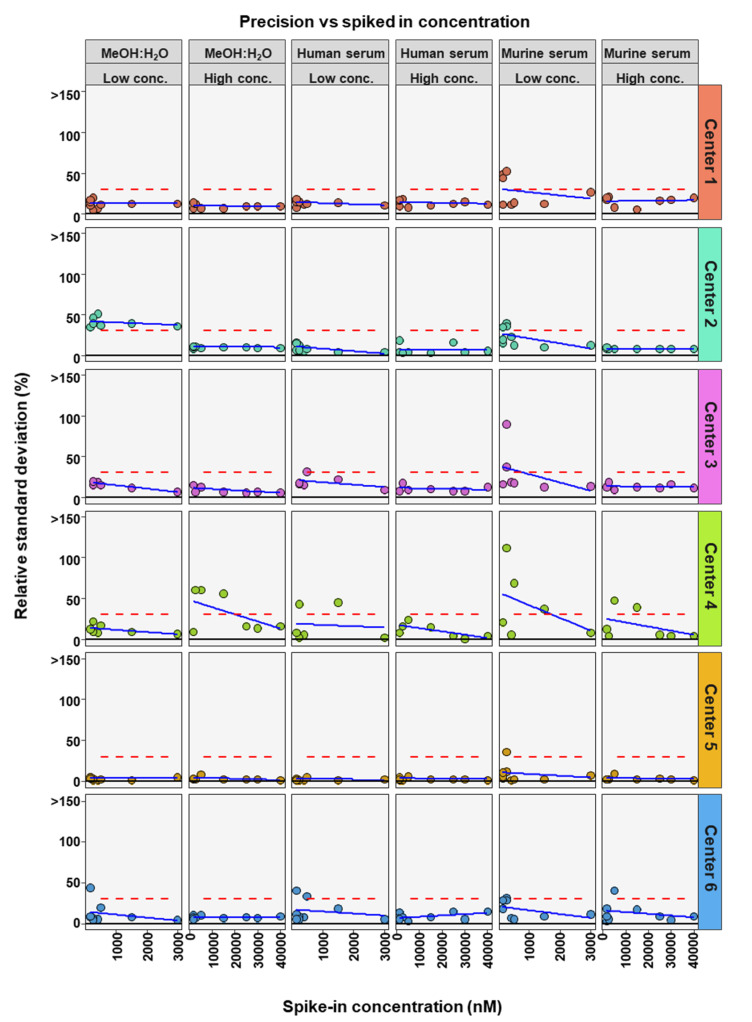
Comparison of spiked concentration of bile acids to precision of measurements for each assessing center.

**Figure 5:**
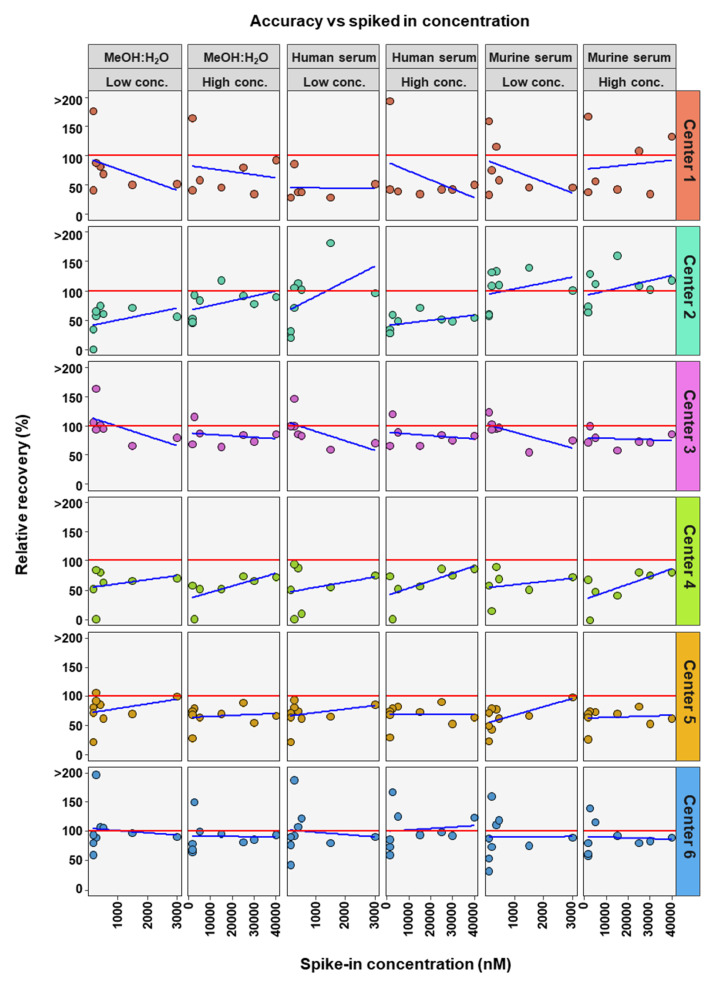
Comparison of spiked concentration of bile acids to accuracy of measurements for each assessing center.

To further determine if BA concentration affects the precision and accuracy of measurements, linear regression analysis was performed comparing either RSD or relative recovery with BA concentrations. This was done for each matrix and high or low concentration range for each center (Figure 4 and Figure 5). Precision was seen to either remain in RSD limits or improve markedly with higher concentrations of BA (Figure 4). For relative recovery versus BA concentration analysis, a more differentiated picture emerged (Figure 5). In most cases accuracy remained within limits over the concentration range, for both high and low concentration spiked samples, values were more spread out at the bottom of each concentration range, respectively, with relative recovery values convergent with increasing concentration.

**Figure 6:**
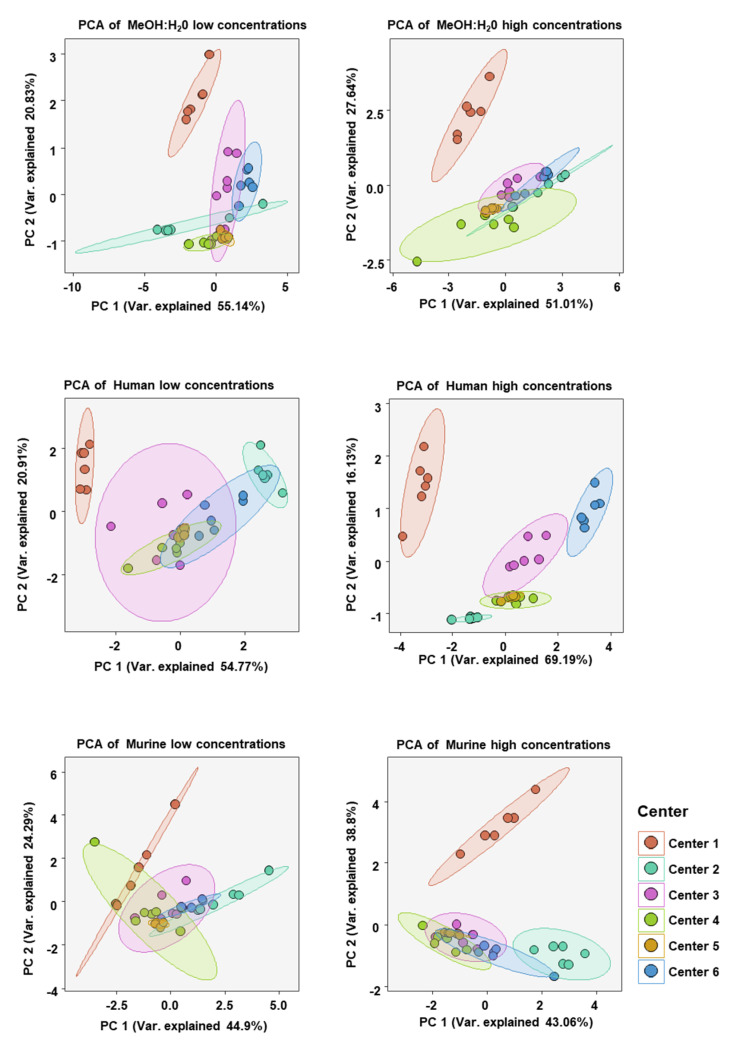
Principal component analysis (PCA) of bile acid concentration profiles measured at each assessing center in either the MeOH:H_2_O solvent, human serum, or murine serum at either high or low bile acid concentrations. BAs included in the analysis were only those which were measurable in all centers (CA, CDCA, DCA, TCA, GCA, β-MCA).

To analyze the similarity of BA concentration profiles between assessing centers we performed principal component analysis (PCA). Only values from BAs which could be measured in all centers were chosen. PCA was done for each matrix at high and low BA concentration spike-ins (see Figure 6). As expected, the BA profiles of each sample at either high or low concentration in each matrix clustered well together. There was also overlapping of various centers with 95% confidence areas, suggesting BA profiles, in general, being relatively similar. One exception was that in the human serum at high BA concentrations there was little overlap of confidence areas, suggesting a greater dissimilarity between the centers than in the other five analyses.

**Figure 7:**
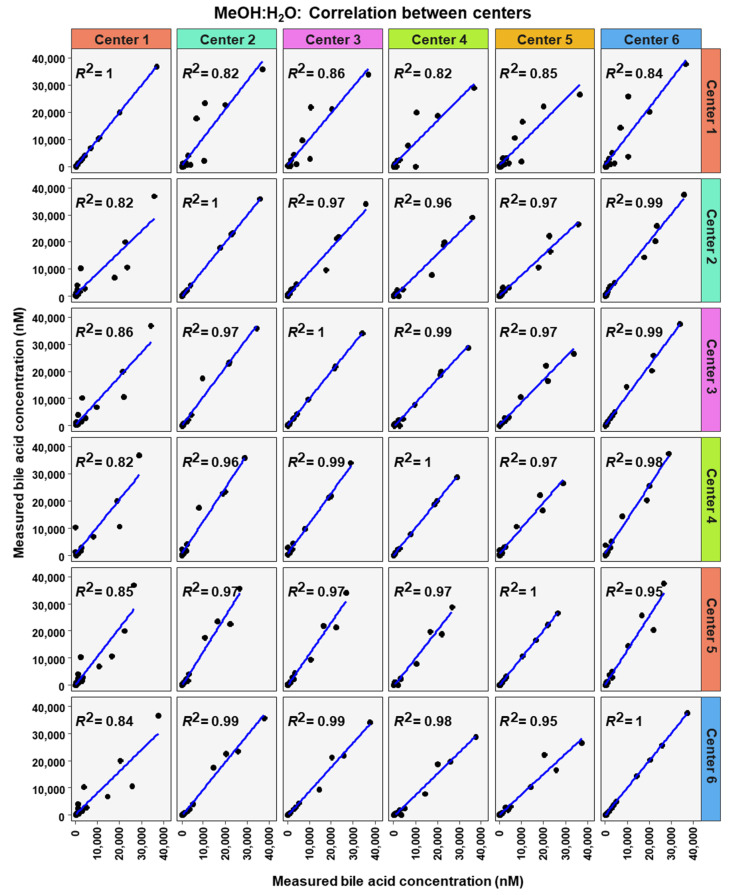
Correlation of bile acid quantification between assessing centers for measurements of bile acid in MeOH:H_2_O.

**Figure 8:**
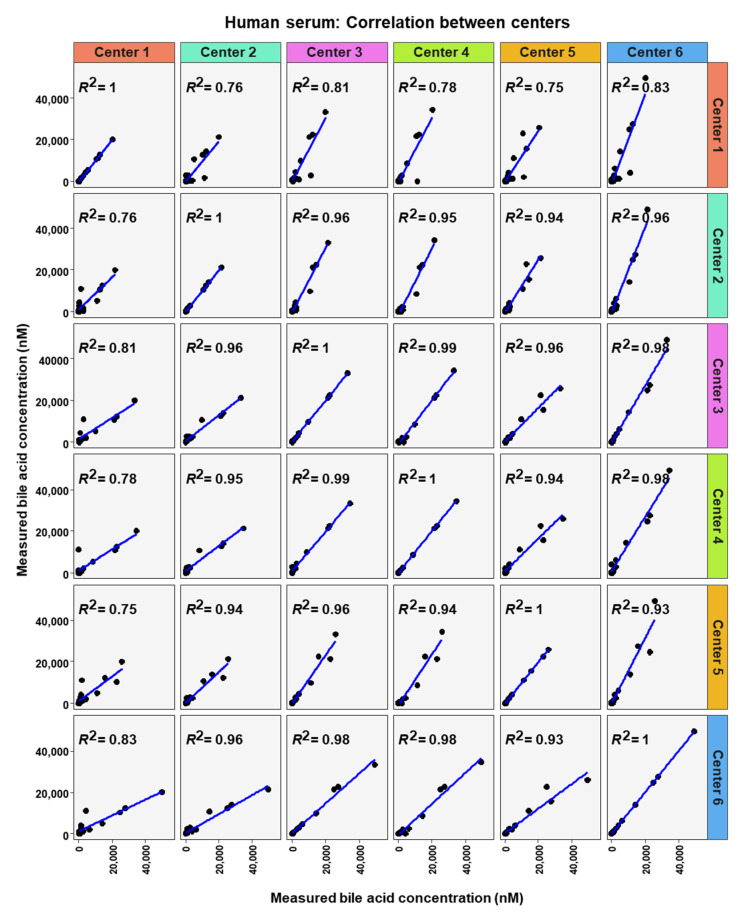
Correlation of bile acid quantification between assessing centers for measurements of bile acid in human serum.

**Figure 9:**
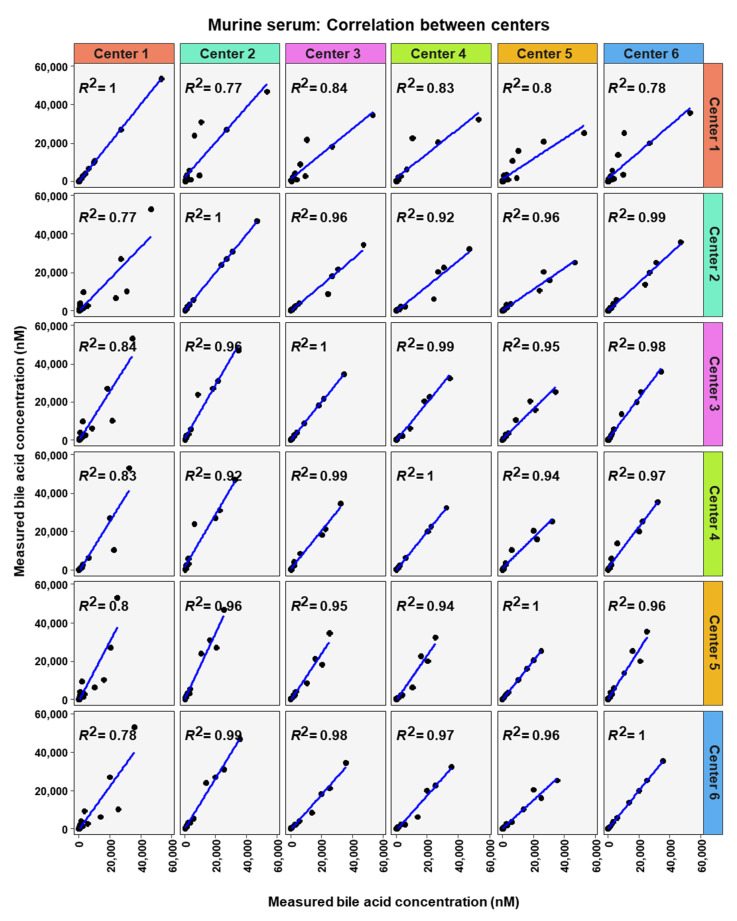
Correlation of bile acid quantification between assessing centers for measurements of bile acid in murine serum.

To analyze similarity in bile acid profiles between the centers in more detail, correlation analysis using linear regression analysis was done for each center pairing and each matrix (Figure 7, Figure 8 and Figure 9). The values from the low and high BA concentration spike-in were combined. For human serum, ten comparisons between centers showed excellent correlations. These were center 2 with center 3 (R^2^ = 0.96), center 2 with center 4 (R^2^ = 0.95), center 2 with center 5 (R^2^ = 0.94), center 2 with center 6 (R^2^ = 0.96), center 3 with center 4 (R^2^ = 0.99), center 3 with center 5 (R^2^ = 0.96), center 3 with center 6 (R^2^ = 0.98), centers 4 with centers 5 (R^2^ = 0.94), center 4 with center 6 (R^2^ = 0.98), and center 5 with center 6 (R^2^ = 0.93). For murine serum, there were also ten pairings with excellent correlation scores with these being the same pairings as for the human serum samples. These were center 2 with center 3 (R^2^ = 0.96), center 2 with center 4 (R^2^ = 0.92), center 2 with center 5 (R^2^ = 0.96), center 2 with center 6 (R^2^ = 0.99), center 3 with center 4 (R^2^ = 0.99), center 3 with center 5 (R^2^ = 0.95), center 3 with center 6 (R^2^ = 0.98), center 4 with center 5 (R^2^ = 0.94), center 4 with center 6 (R^2^ = 0.97), and center 5 with center 6 (R^2^ = 0.96).

## 3. Discussion

Apart from a multicenter ring trial assessing the robustness of a commercially available bile acid kit for LC-MS/MS-MRM [22], there is to our knowledge no previous multi-center ring trial assessment of targeted LC-MS/MS methods for the quantification of bile acids in serum samples. In this ring trial assessment, centers were recruited based on the partners connected to third party-funded multicenter projects (SFB1382; “Gut–liver axis” and SFB1371 “Microbiome Signatures”).

Overall, it was found that all centers are able to determine bile acids with different methods (see summary in Table 4) from different matrices in absolute quantities with high quality.

With respect to the applied methods, five of the six participating centers used methods partly adapted from the literature, while one center (center 6) used a commercial kit-based technology. Centers 1 and 5 referred to a method by García-Cañaveras et al. [23], while center 2 worked with a method by Amplatz et al. [24]. The method used by center 3 is based on a paper by Reiter et al. [25] and the assay established by center 4 is based on the paper by Tagliacozzi et al. [26]. Center 6 used the commercial kit-based method, which was first described in 2016 [22]. The original publications contain additional information such as example chromatograms showing the analytical separation of the individual bile acids, details of the MRM transitions used, and information on the quantification ranges.

MRM methods on triple quadrupole instruments are regarded as the gold standard for the targeted detection of small molecules [27]. For the mass spectrometry assessment, five out of six groups used MRM triple quadrupole-based methods. Two different quadrupole devices were used. One was a Q-Trap system from Sciex (4 cases) and the other was the Xevo TQ-S system from Waters. An advantage of triple quadrupole instruments is the broad linear dynamic range of detection and higher sensitivity. Only one group (center 4) used a high-resolution mass spectrometer for the analyses (Q Exactive™ mass spectrometer (Thermo Fisher Scientific, Waltham, MA, USA) with a quadrupole precursor selection and high-resolution Orbitrap™ detection). The general disadvantage that this can only cover a smaller linear dynamic range and lower sensitivity than low-resolution instruments has not significantly affected the results obtained with the instrument. On the other hand, the higher mass resolution allows better discrimination of molecules with similar molecular weights and may help in distinguishing bile acids. However, a number of bile acids have the same molecular weight, such as CDCA and DCA, or α-MCA, β-MCA, and ω-MCA. We observed no great difference between analyses with the quadrupole instruments and the orbitrap instrument.

In all methods, the mass spectrometers were coupled to LC systems. Centers 1 and 4 analyzed their samples using a combination of an ACQUITY UPLC coupled with a Xevo TQ-S system (Waters GmbH, Eschborn, Germany). Three centers (3, 5, and 6) all used QTRAP 5500 (Sciex, Toronto, ON, Canada) mass spectrometers coupled to different LC systems. Center 3 used an ExionLC AD UPLC system (Sciex, Darmstadt, Germany). Center 5 worked with a 1290 Infinity UPLC system (Agilent Technologies, Santa Clara, CA, USA) and Center 6 used an ACQUITY UPLC system (Waters GmbH, Eschborn, Germany). Except for center 4, all participants used electrospray ionization in negative ion mode. Center 4 ionized using a Unispray source, though this ionization source includes an electrospray ionization mode.

Despite the similarities in the detection, significant differences were striking with respect to the sample volume required for bile acid extraction and measurement, with a range between 10 µL–50 µL. This difference can be critical for studies where sample volumes are an issue e.g., portal vein blood from mice. Therefore, methods requiring larger volumes may be limited in their applicability to certain studies. The lowest sample volumes were used in centers 2 and 6 (10 µL,) followed by centers 1, 3, and 5, which require (25 µL, 30 µL, and 50 µL) for their method, respectively. In general, more stable results can be obtained when using higher sample quantities. Fortunately, the centers that used lower amounts of material also achieved stable and good results.

All centers performed protein precipitation on the samples before doing further analytical steps. Further details of sample preparation differed between centers and can be found in the methods section. A limiting step is the number of samples, which can be measured in an acceptable amount of time, is the length of the LC method. The HPLC methods varied greatly between centers. Depending on the column and gradient used, the runs lasted between 5 (center 6) and 25 min (centers 2 and 3). The detailed methods are described below. Interestingly, this difference in LC-method duration did not alter the outcome of the bile acid quantification results. All centers worked with internal standards and applied in-house quality control steps to assure the best possible identification and quantification results. In one case (kit-based method), there was a software-supported solution for quality control. It is worth mentioning that center 3 subjected all reference substances used for the calibration to prior quality control by quantitative NMR measurement (Bruker AVANCE III 400 MHz system Bruker, Rheinstetten, Germany).

One important observation in this study was that acceptable precision, which reached 261 times out of 300 cases, was more often achieved than acceptable recovery, 143 times out of 300 cases. Interestingly, the recovery levels were usually below 100% and the ratio between high concentration and low concentration of bile acid in each matrix was often very close to the theoretical value for many of the BAs. Taken together this suggested that most of the error in concentration was not due to the LC-MS/MS methods, but probably occurred before. A number of parameters could contribute to the error, for example, deterioration of samples during transportation or loss of BAs during sample preparation, which included protein precipitation and solid-phase extraction. Most likely, the sample protein precipitation and solid-phase extraction did not contribute significantly to the error since internal standards were added to the samples during protein precipitation. In addition, sample handling has not been identified as a major error source [28]. Center 1 BA concentrations differed the most when centers were compared (Figure 7 and in the correlation analysis between centers in Figure 7, Figure 8 and Figure 9. However, center 1 precision of measurements was similar to the other centers. These facts lead us to assume that a deterioration of the samples for center 1 during transportation occurred. Acceptable precision was slightly more often reached in the MeOH:H_2_O matrix than for either murine or human serum samples. A possible reason for this is that in blood BAs are usually bound to albumin [29,30], therefore when extracting BAs from serum samples they must be released from albumin.

From the results of the recoveries, it can be concluded that bile acids can be ionized differently depending on their structure. In principle, bile acids with higher polarity/higher hydrophilicity and conjugated BAs can be ionized better. One analytical challenge in the detection of bile acids is that some of the bile acids like CDCA or LCA do not fragment well which is why often only the precursor ion is detected in the MRM-mode. One possible explanation for this is that it is very difficult to obtain stable fragments for unconjugated bile acids since for conjugated BAs the glycine or taurine moiety can be cleaved and measured as a stable fragment. For this reason, measurement in pseudo-selected monitoring mode is used for these unconjugated BAs. In this mode, the parent and the daughter ions have the same mass. This is achieved by working with low collision energy that does not lead to any fragmentation of the parent ion but at the same time leads to an improvement of the signal-to-noise ratio [31]. In our study, as expected, CA, a hydrophilic BA, and its two conjugates GCA and TCA were the three BAs most often within recovery limits (CA (24 times) and its conjugates GCA (28 times) and TCA (31 times)). In contrast, LCA (4 times) and ω-MCA (1 time), both unconjugated hydrophobic BAs, performed worst. 

The results reveal that the accuracy based on relative recovery was similar for measurements at low or high concentrations of BAs (number of BA analyses within relative recovery limits: maximum 25, minimum 22), which can be seen for the generally good stability of the measurements in all centers.

The excellent correlation of the data between the individual centers (from 0.82 to 0.99), revealed that quantification results can be compared between the assessing centers in this study. This suggests that data obtained from different centers can be used for e.g., pooled analysis.

We are aware of some shortcomings in this study. One of them is that we did not analyze the effects of the various sample preparation methods here. But this is the subject of an ongoing study in the framework of an international ring trial, in which also some partners of this group are involved. A further limiting factor of this study was the low number of participating assessment centers. It is known that many academic institutions and commercial laboratories measure BAs in blood samples. We are aware of the situation that commercial labs are required to pass official ring trials on a regular basis. However, this study was focused on the absolute quantification of BAs in assessment centers involved in the aforementioned third party-funded project (SFB 1382, SFB 1371), and was the first attempt to compare methods from different centers. It could be an impulse for academic BA analysis laboratories to use ring trials to assess their methods in regard to accuracy and reproducibility. In the future, other BAs could be incorporated into a ring trial, including recently discovered novel BAs. Recently, bile acids coupled to amino acids other than taurine and glycine have been identified. Quinn et al. [32] found novel BAs of cholic acid conjugated to non-canonical amino acids like phenylalanine, tyrosine, and leucine. It has been shown that additional amino acids are coupled to bile acids by microbes [33,34,35]. These novel conjugated bile acids have been shown in in vitro experiments to bind to receptors [32], and therefore are an interesting candidate to be included in bile acid quantification assays.

## 4. Materials and Methods

### 4.1. Sample Preparation for the Ring Trial

A central independent lab (that was not actively taking part in the analytical procedures or data analysis) prepared test samples for all assessing centers using the following nine BAs: LCA (CAS No. 434-13-9, European Pharmacopoeia, Strasbourg, France), CDCA (CAS No. 474-25-9, European Pharmacopoeia), TCA (CAS No. 145-42-6, MP Biomedicals, Eschwege, Germany), CA (CAS No. 81-25-4, United States Pharmacopeia, Washington DC, USA), DCA (CAS No. 83-44-3, United States Pharmacopeia), GCA (CAS No. 330277W, Avanti Inc., Alabaster, AL, USA), α-MCA (CAS No. 2393-58-0, Avanti Inc. Alabaster), ω-MCA (CAS No. 700231, Avanti Inc. Alabaster), and β-MCA (CAS No. 2393-59-1, Sigma-Aldrich Chemie GmbH, Taufkirchen, Germany). For sample preparation, all pipetting steps were performed on ice, sample tubes were closed as quickly as possible to limit evaporation. First, stock solutions were prepared at 10 mg/mL using HPLC-grade methanol as solvent. Next, BAs were mixed into standard diluent/DIL (MeOH:H_2_O, 1:1, *v*/*v*) at fixed concentrations to create a master mix for high concentrations (MM_HI_). Concentrations for MM_HI_ were calculated so they would yield the desired final concentrations (high, Table 1) when 191 µL of the mix were diluted in 3.5 mL of matrix. The master mix for low concentrations (MM_LO_) was produced by diluting BA stocks either 1:100 (TCA, GCA) or 1:10 (all other BAs) and then mixing these dilutions into DIL to yield the desired concentrations. Concentrations for MM_LO_ were calculated so they would yield the desired final concentrations (low, Table 1) when 91.3 µL of the mix was diluted in 3.5 mL of matrix. Three different matrices were used: a pool of human sera (acquisition and usage approved by the local Ethics committee, University of Aachen, EK 025/19, to T.C.), commercially available murine serum (catalog No. S7273, Sigma-Aldrich Chemie GmbH, Taufkirchen, Germany) and standard diluent/DIL (MeOH:H_2_O, 1:1). Spiking MM_HI_ (191 µL/3.5 mL) or MM_LO_ (91.3 µL/3.5 mL) into the three matrices resulted in six master samples with either high concentration or low concentration of all nine BAs. Pooled human serum and murine serum were included as additional sample types. Aliquots of 50 µL or 100 µL (depending on requests by the centers) were prepared in precooled tubes and stored in cryoboxes at −80 °C. Sample boxes were all sent out on the same day by courier on dry ice. Each of the six assessing centers (Centers 1–6) received a box with six analytical replicates each of the human serum, the murine serum, and the solvent spiked with the bile acids at either high or low concentrations plus six analytical replicates of each of the human and murine serum without the spiked BAs (to be used for determining baseline levels of BAs in the matrices). While the centers were told the concentration range of the samples, the exact concentration of each BA was only known to the independent lab.

After the assessing centers measured the samples they reported their results to the central independent laboratory, which blinded the results (using aliases for assignment, i.e., ‘center 1–6′) and sent them on to the data team for analysis without disclosing the centers’ identities. 

### 4.2. Center 1: Sample Preparation, LC-MS/MS, and Raw Data Analysis

#### 4.2.1. Background

LC-MS/MS analysis of bile salt composition followed the methodology of García-Cañaveras et al. [23], with modifications that allowed full separation of α-, β-, and ω-muricholic acid and their taurine conjugates, and concomitant quantification of 7α-hydroxy-4-cholesten-3-one, a serum marker reflecting bile salt synthesis.

#### 4.2.2. Materials, Calibration Standards and Quality Control

All solvents were of UPLC/MS grade. Stock solutions of individual bile salts were prepared in methanol and combined to yield a standard solution. A 12-point calibration curve (final concentration: 0.3 to 1250 nmol/L for each species) was prepared by serial dilution in methanol. Standards were spiked in human citrate plasma to enhance endogenous levels with the same range of concentrations, to determine recovery. Endogenous bile salt levels in the employed plasma pool were used to monitor the between-run consistency of the method.

A total of 9 deuterated bile salts representative of the studied classes and with elution times distributed along the chromatographic run were used as internal standards and dissolved in methanol. The final concentration in processed samples was 100 nmol/L for each internal standard.

#### 4.2.3. Sample Preparation

Samples (25 µL) were deproteinated with 2 volumes of methanol and 1 volume of internal standard solution in screw cap vials. Following vigorous vortexing (3 × 10 s), samples were centrifuged (15 min 50,000× *g*, 4 °C) and the supernatant was transferred to glass micro-insert vials that were closed using a silicon rubber seal.

#### 4.2.4. Equipment

Sample supernatants (5 µL) were injected using a PAL3 RSI sample processor onto an ACQUITY UPLC BEH Shield RP18 column (Waters, Mississauga, ON, Canada; particle size: 1.7 µm, length: 100 mm, internal diameter: 2.1 mm). The initial solvent was 85% A (solvent A: 95% H_2_O, 5% acetonitrile containing 10 mmol/L ammonium acetate, solvent B: 100% acetonitrile) at a flow rate of 0.7 mL/min at 50 °C. Gradient elution (Table 5) was performed using an UltimateTM 3000 quaternary UPLC pump (Thermo Scientific, Waltham, MA, USA). Mass spectrometric detection employed a Xevo TQ-S (Waters) with electrospray ionization in negative ion mode. Individual bile salts were monitored in MRM windows, either as pseudo-transitions (for unconjugated species) or as glycine/taurine daughter ions.

#### 4.2.5. Data Analysis

The data were processed using Target Lynx v4.1. All peaks were visually inspected for correct integration, after which data were exported to Excel. AUC values were corrected for recovery of the assigned internal standard. Unweighted linear regression was used to calculate concentrations from standard curves. Responses of individual bile salt species were linear up to at least the highest test concentration of 100 µmol/L.

### 4.3. Center 2: Sample Preparation, LC-MS/MS, and Raw Data Analysis

#### 4.3.1. Background

LC-MS/MS analysis of bile salt composition followed the methodology of Amplatz et al. [24], with modifications. Individual BAs were separated by HPLC using a reversed-phase C18 column (Kinetex C18, Phenomenex, Aschaffenburg, Germany). Quantification and characterization were achieved using a Q Exactive™ mass spectrometer (Thermo Fisher Scientific, Waltham, MA, USA) with a quadrupole precursor selection and high-resolution (HR/AM) Orbitrap™ detection.

#### 4.3.2. Calibration Standards and Quality Control

Stock solutions of individual bile salts were prepared in methanol and combined to yield a standard solution. A 10-point calibration curve (final concentration: 4.9 to 2500 nmol/L for each species) was prepared by serial dilution in methanol. Standards were spiked in human and murine serum to determine recovery. A total of 6 deuterated bile salts representative of the studied classes and with elution times distributed along the chromatographic run, were used as internal standards and dissolved in methanol. Unconjugated CA, CDCA, DCA, LCA, as well as their T- and G-conjugates; alpha, beta, and omega-muricholic acid (MCAs) and T- MCAs, as well as internal standards d4-DCA, d4-LCA, d4-glyco-LCA (d4-GLCA) and d4-glyco-CDCA (d4-GCDCA), d4-tauro-CA, DCA (d4-TCA, d4-TDCA), (all Sigma Aldrich, Taufkirchen, Germany) and alpha, beta and omega-MCA (Steraloids, Newport, RI), were used for identification and quantification in MS analysis. 

#### 4.3.3. Sample Preparation

Both murine and human plasma or serum samples were prepared in the same way. After the addition of the internal standards (0.2 nmol each), the samples (10 μL) were vortexed for 1 min. 400 μL of acetonitrile was added for deproteinization. After vortexing and centrifugation at 3200× *g* for 12 min at RT, the supernatant was removed and dried under a stream of nitrogen at 50 °C. The samples were redissolved in 100 μL of mobile phase B and transferred to autosampler vials.

#### 4.3.4. Equipment

Samples (10 μL), stored in a cooled stack (Thermo Fisher Scientific) were introduced into the chromatographic system by an autosampler (Accela Open AS, Thermo Fisher Scientific). A Kinetex C18 reversed phase column (2.6 μm, 100 × 3.0 mm) (Phenomenex, Aschaffenburg, Germany) mounted in an oven with a column-switching unit (Mistraswitch, Maylab, Vienna, Austria, set to 25 °C) which was used for HPLC of human BA samples. The HPLC pump was a 1250 Accela (Thermo Fisher Scientific). A gradient of mobile phase A (aqua dest. with 1.2% *v*/*v* formic acid and 0.38% *w*/*v* ammonium acetate) and eluent B (acetonitrile with 1.2% *v*/*v* formic acid and 0.38% *w*/*v* ammonium acetate) was used for separation and elution. Gradient settings for eluents are listed in Table 6. The flow rate was 500 μL/min. A Q Exactive hybrid quadrupole-orbitrap mass spectrometer (Thermo Fisher Scientific) was used with a heated ESI ion source with negative ionization. The settings used for ionization were: sheath gas flow rate 40 mL/min, auxiliary gas flow rate 10 mL/min, sweep gas flow rate 0 mL/min, spray voltage 3.00 kV, capillary temperature 380 °C, S-lens RF (radio frequency) level 50, and auxiliary gas heater temperature 450 °C. Negative ion full scan mode was set between *m*/*z* = 370 to *m*/*z* = 530; resolution was 70,000 (specified at *m*/*z* = 200). Formic acid adducts of unconjugated BAs were taken into analysis in all qualitative and quantitative experiments.

#### 4.3.5. Data Analysis

Linear calibration (weighting 1/×2) was done by correlation of peak area ratios of natural targets vs. internal standards of 10 diluted (1:1) standard samples in NaCl (0.9% in aqua dest.) with known amounts in a range of 0.049 pmol/sample to 2.5 pmol/sample in double estimation for all targets. Xcalibur 2.3 software (Thermo Fisher Scientific) was used for instrument control as well as for the calculation of target concentrations.

### 4.4. Center 3: Sample Preparation, LC-MS/MS, and Raw Data Analysis

#### 4.4.1. Background

LC-MS/MS analysis of bile acids (BA) was carried out according to Reiter et al. [25].

#### 4.4.2. Materials, Calibration Standards and Quality Control

All solvents were of LC-MS grade. Prior to the preparation of calibration curves, reference substances were weighed in 5 × 178 mm NMR tubes (USC tubes, Bruker, Faellanden, Switzerland) and solved in methanol-d4. 1H qHNMR analyses of each bile acid was done on a Bruker AVANCE III 400 MHz system Bruker, Rheinstetten, Germany) equipped with a Z-gradient 5 mm multinuclear observe probe (BBFOplus) at 298 K and Topspin 3.6 software to determine the accurate concentration and purity of stock solutions using ERETIC II, as described by Frank et al. [36]. Based on qHNMR data, BA and internal standards were diluted with methanol to receive individual stock solutions of 1 mmol/L and stored at −80 °C until use.

The solutions of the eight deuterated internal standards were combined and diluted with methanol to get an internal standard mix containing approximately 7 μmol/L of each deuterated bile acid.

For the calibration curves, stock solutions of all bile acids were pooled together and diluted successively with methanol and the internal standard solution was added to obtain concentrations of 0.5 nmol/L to 15,000 nmol/L for the analytes and 250 nmol/L for the isotopically labeled standards. After analysis, the calibration curves for all 45 bile acids are generated by plotting the peak area ratios of analyte to internal standard against the concentration ratios of analyte to internal standard using linear regression using 1/× weighting.

#### 4.4.3. Sample Preparation

Samples (30 µL) were diluted with a methanol-based dehydrocholic acid (DHCA) extraction solvent (270 µL, 1.3 µmol/L) as an internal standard accounting for work-up loss. After shaking (15 min, 1000 rpm, 10 °C), samples were centrifuged (10 min, 8000 rpm, 4 °C). The supernatant (100 µL) was spiked with the internal standard mix (20 µL) and transferred to an autosampler vial equipped with a glass insert.

#### 4.4.4. Equipment

Samples were analyzed using an LC-MS/MS system consisting of a QTRAP 5500 triple quadrupole mass spectrometer (Sciex, Darmstadt, Germany) coupled to an ExionLC AD (Sciex, Darmstadt, Germany) ultrahigh performance liquid chromatography system consisting of two LC pump systems ExionLC AD, an ExionLC degasser, an ExionLC AD autosampler, an ExionLC AC column oven, and an ExionLC controller. A multiple reaction monitoring (MRM) method was used for the detection and quantification of BA. For separation of these compounds, a 100 × 2.1 mm, 100 Å, 1.7 μm, Kinetex C18 column (Phenomenex, Aschaffenburg, Germany) and a SecurityGuard™ ULTRA Cartridges UHPLC C18 2.1 mm column (Phenomenex, Aschaffenburg, Germany) was used. The chromatography was performed with a column temperature of 40 °C and a constant flow rate of 0.4 mL/min using the mobile phase consisting of eluent (A) water and eluent (B) acetonitrile/water (95/5, *v*/*v*), both containing 5 mM ammonium acetate and 0.1% formic acid. The gradient elution is described in Table 7. The injection volume for all samples was 1 μL, the column oven temperature was set to 40 °C, and the autosampler was kept at 10 °C. Data acquisition and instrumental control were performed with Analyst 1.7 software (Sciex, Darmstadt, Germany).

#### 4.4.5. Data Analysis

Data were processed and analyzed using MultiQuant v3.0.3 (Sciex, Darmstadt, Germany). All peaks were manually checked for correct integration and the data were then exported to Excel. BA concentrations were corrected for work-up loss via the internal standard DHCA and expressed as nmol/L.

### 4.5. Center 4: Sample Preparation, LC-MS/MS, and Raw Data Analysis

#### 4.5.1. Background

LC-MS/MS analysis of bile salt composition followed the methodology by Tagliacozzi et al. [26], with modifications that allowed the separation of α/ω- and β-muricholic acid and their taurine conjugates.

#### 4.5.2. Materials, Calibration Standards and Quality Control

All solvents were of UPLC/MS grade. Stock solutions of individual bile salts were prepared in methanol and combined to yield a standard solution. A 12-point calibration curve (final concentration: 0.3 to 1250 nmol/L for each species) was prepared by serial dilution in methanol. Standards were spiked in human clinical chemistry control serum to enhance endogenous levels with the same range of concentrations, to determine recovery. Endogenous bile salt levels in the employed plasma pool were used to monitor the between-run consistency of the method.

A total of 9 deuterated bile salts representative of the studied classes from CDN (Quebec, Canada) and Toronto Research Chemicals (Downsview, ON, Canada) (d4-LCA, d4-UDCA, d5-CA, d4-GLCA, d4-GDCA, d4-GCDCA, d4-GUDCA, d4-GCA, d4-TCA; each of 25 ng/40 µL methanol) and with elution times distributed along the chromatographic run were used as internal standards and dissolved in methanol. 

#### 4.5.3. Sample Preparation

Samples (100 µL) were deproteinated with 800 µL acetonitrile and 40 µL of internal standard solution in screw cap vials. Following vigorous vortexing (3 × 10 s), samples were centrifuged (15 min 13.000× *g*, room temp.) and the supernatant was evaporated and reconstituted in 240 μL methanol:water (1:1) with stepwise addition and vortexing of the solvents. 

#### 4.5.4. Equipment

Sample supernatants (5 µL) were injected using a Waters FTN Sample manager onto an ACQUITY UPLC BEH Shield RP18 column (Waters; particle size: 1.7 µm, length: 50 mm, internal diameter: 2.1 mm). The initial solvent was 81% A (solvent A: 100% water with 5 mmol/L ammonium acetate and 0.01% formic acid; solvent B: 100% methanol with 5 mmol/L ammonium acetate and 0.01% formic acid) at a flow rate of 0.4 mL/min at 45 °C. Gradient elution (Table 8) was performed using a Waters ACQUITY UPLC I-class pump. Mass spectrometric detection employed a Xevo TQ-XS (Waters) with Unispray ionization in negative ion mode. Individual bile salts were monitored in MRM windows, either as pseudo-transitions (for unconjugated species) or as glycine/taurine daughter ions.

#### 4.5.5. Data Analysis

Data were processed using Target Lynx v4.1. All peaks were visually inspected for correct integration, after which data were exported to Excel. AUC values were corrected for recovery of the assigned internal standard. Unweighted linear regression was used to calculate concentrations from standard curves. Responses of individual bile salt species were linear up to at least the highest test concentration of 100 µmol/L.

### 4.6. Center 5: Sample Preparation, LC-MS/MS, and Raw Data Analysis

#### 4.6.1. Background

LC-MS/MS analysis of bile salt composition followed the methodology of García-Cañaveras et al. [23], with modifications that allowed full separation of α-, β-, and ω-muricholic acid and their taurine conjugates, and concomitant quantification of 7α-hydroxy-4-cholesten-3-one, a serum marker reflecting bile salt synthesis.

#### 4.6.2. Materials, Calibration Standards and Quality Control

All solvents were of LC-MS grade. Stock solutions of individual bile salts were prepared in methanol and combined to yield a standard solution. A 12-point calibration curve (final concentration: 0.3 to 1250 nmol/L for each species) was prepared by serial dilution in methanol. Standards were spiked in human clinical chemistry control serum to enhance endogenous levels with the same range of concentrations, to determine recovery. Endogenous bile salt levels in the employed plasma pool were used to monitor the between-run consistency of the method.

A total of 10 deuterated bile salts representative of the studied classes from CDN isotopes (Quebec, Canada, d4-GCDCA, d4-GLCA, d4-GCA, d4-GUDCA, d4-CDCA, d4-UDCA, d4-LCA) and Toronto Research Chemicals (Downsview, Ontario, Canada, d4-TCA, d6-C4) and with elution times distributed along the chromatographic run, were used as internal standards and dissolved in methanol. The final concentration in processed samples was 100 nmol/L for each internal standard.

#### 4.6.3. Sample Preparation

Samples (50 µL) were deproteinated with 500 µL internal standard solution in methanol in screw cap vials. Following vigorous vortexing (3 × 10 s), samples were centrifuged (15 min 50,000× *g*, 4 °C) and the supernatant was evaporated and reconstituted in 200 μL methanol:water (1:1) of which 150 μL were transferred into autosampler vials.

#### 4.6.4. Equipment

Sample supernatants (5 µL) were injected using a PAL3 RSI sample processor onto Kinetex C18 column (2.1 × 100 mm with 1.7 μm particles) (Phenomenex, Torrance, CA, USA) and kept at 60 °C. The initial solvent was 80% A (solvent A: 100% H2O, 7.5 mM ammonium acetate, and 0.019% formic acid at a pH of 4.5, solvent B: 100% acetonitrile with 0.1% formic acid) at a flow rate of 0.4 mL/min. Gradient elution (Table 9) was performed using a 1290 Infinity quaternary UPLC pump (Agilent Technologies, Santa Clara, CA, USA). Mass spectrometric detection employed a QTRAP 5500 (Sciex, Toronto, Canada) with electrospray ionization in negative ion mode. Individual bile salts were monitored in MRM windows, either as pseudo transitions (for unconjugated species) or as glycine/taurine daughter ions.

#### 4.6.5. Data Analysis

Data were processed using Sciex Multiquant 3.0.3. All peaks were visually inspected for correct integration, after which data were exported to Excel. AUC values were corrected for recovery of the assigned internal standard. Unweighted linear regression was used to calculate concentrations from standard curves. Responses of individual bile salt species were linear up to at least the highest standard concentration of 3000 nmol/L.

### 4.7. Center 6: Sample Preparation, LC-MS/MS, and Raw Data Analysis

#### 4.7.1. Background

For the bile acid determination, the Bile acid Kit from Biocrates (Innsbruck, Austria) was used. The exact description of the procedure can be found in Phamet al. [22]. 

#### 4.7.2. Materials, Calibration Standards and Quality Control

All solvents were of LC-MS grade. 10 µL of standards and internal standards mixture was pipetted onto the filter spots suspended in the wells of the 96-well filter plate. The filter plate was fixed on the top of a deep-well serving as a receiving plate for the extract (a combi-plate structure). In addition, quality controls were distributed on the plate. On the one hand, one QC each was measured with low, medium, and high concentrations of the calibration range, and additionally 3 further medium QC samples were distributed over the plate. A 7-point calibration curve (concentration range: 10 to 75,000 nmol/L bile acid-specific) was used for calibration.

#### 4.7.3. Sample Preparation

10 µL samples were pipetted on the spots of the kit plate, followed by nitrogen drying. Then 100 µL methanol was added to the wells, and the combi-plate was shaken for 20 min. The combi-plate was centrifuged to elute the methanol extract into the lower receiving deep-well plate, which was then detached from the upper filter plate. After adding 60 µL Milli-Q water to the extracts, the samples were analyzed.

#### 4.7.4. Equipment

5 µL of the sample was injected using ACQUITY UPLC System (Waters) (UHPLC Column from Biocrates P.-No 91220052120868). The initial solvent was 65% A (solvent A: 100% H2O, containing 10 mmol/L ammonium acetate), solvent B: 100% acetonitrile containing 10 mmol/L ammonium acetate and 0.1% formic acid) at a flow rate of 0.5 mL/min at 50 °C. Gradient elution (Table 10) was performed using a Q-Trap 5500 mass spectrometer (Sciex). Mass spectrometric detection was employed with electrospray ionization in negative ion mode (IS-4500 eV). Individual bile salts were monitored in MRM windows.

#### 4.7.5. Data Analysis

Data were processed using Analyst 1.7.1. All peaks were visually inspected for correct integration including the internal standards using the quantification software from Analyst. AUC values were corrected for recovery of the assigned internal standard. Quadratic regression was used to calculate concentrations from standard curves. Afterward, further quality control was carried out using the Met IDQ software (version Oxygen) provided by the Biocrates company.

### 4.8. Statistical Data Analysis

Data containing the bile acid concentrations in all samples were blinded and sent to the data analysis team. Centers were given IDs 1 to 6 to keep anonymity. Missing values for bile acid concentrations were regarded as true missing values. The exception was that if in all six replicate measurements these were missing values, then this was regarded as zero values. The bile acid concentration values determined in the human or murine serum samples were corrected for residual BA contained in the matrix by subtracting the mean values detected in the six replicate measurements from the non-spiked human and murine serum, respectively. Further data analysis was done in R using in-house written scripts [37]. Precision was determined by calculating the relative standard deviation of each analysis to the mean value, while accuracy was determined by relative recovery, i.e., the mean concentration detected compared to the known concentration spiked into the matrix. Figures were created in R using the ggplot2 package [38].

All centers confirmed that they had only used calibrations in the linear range of the calibration curve or had diluted the samples through the corresponding range and measured them. Afterward, the dilution was back-calculated for the concentration indication.

## 5. Conclusions

The ring trial was a good opportunity to assess the six analytical centers and revealed that by the high correlation between measurements that data sets from different centers can be combined for analysis.

## Figures and Tables

**Table 1:** List of bile acids and concentrations spiked into the matrices.

Bile Acid	High Concentration (nM)	Low Concentration (nM)	Factor (High Conc./Low Conc.)	Bile Acid Class
CA	30,000	3000	10	primary
CDCA	15,000	1500	10	primary
GCA	40,000	400	100	conjugated primary
TCA	25,000	250	100	conjugated primary
DCA	5000	500	10	secondary
LCA	1500	150	10	secondary
α-MCA	1500	150	10	murine primary
β-MCA	2500	250	10	murine primary
ω-MCA	1500	150	10	murine secondary

**Table 2:** Bile acid analyses within precision limits based on relative standard deviation (RSD). NA= not applicable, those BAs are not covered by the specific center.

Center	Matrix	Conc. Level	# BAs in Method	# BAs in RSD Limits	Bile Acid RSD in Limits								
					CA	CDCA	GCA	TCA	DCA	LCA	α-MCA	β-MCA	ω-MCA
Center 1	MeOH:H_2_O	High	9	9	Yes	Yes	Yes	Yes	Yes	Yes	Yes	Yes	Yes
		Low	9	9	Yes	Yes	Yes	Yes	Yes	Yes	Yes	Yes	Yes
	Human serum	High	9	9	Yes	Yes	Yes	Yes	Yes	Yes	Yes	Yes	Yes
		Low	9	9	Yes	Yes	Yes	Yes	Yes	Yes	Yes	Yes	Yes
	Murine serum	High	9	9	Yes	Yes	Yes	Yes	Yes	Yes	Yes	Yes	Yes
		Low	9	5	Yes	Yes	Yes	-	Yes	Yes	-	-	-
Center 2	MeOH:H_2_O	High	9	9	Yes	Yes	Yes	Yes	Yes	Yes	Yes	Yes	Yes
		Low	9	1	-	-	-	-	-	Yes	Yes	-	-
	Human serum	High	9	9	Yes	Yes	Yes	Yes	Yes	Yes	Yes	Yes	Yes
		Low	9	9	Yes	Yes	Yes	Yes	Yes	Yes	Yes	Yes	Yes
	Murine serum	High	9	9	Yes	Yes	Yes	Yes	Yes	Yes	Yes	Yes	Yes
		Low	9	6	Yes	Yes	Yes	-	Yes	Yes	Yes	-	-
Center 3	MeOH:H_2_O	High	7	7	Yes	Yes	Yes	Yes	Yes	NA	Yes	Yes	NA
		Low	7	6	Yes	Yes	Yes	Yes	Yes	NA	-	Yes	NA
	Human serum	High	7	7	Yes	Yes	Yes	Yes	Yes	NA	Yes	Yes	NA
		Low	7	5	Yes	Yes	Yes	Yes	-	NA	-	Yes	NA
	Murine serum	High	7	7	Yes	Yes	Yes	Yes	Yes	NA	Yes	Yes	NA
		Low	7	5	Yes	Yes	Yes	-	Yes	NA	Yes	-	NA
Center 4	MeOH:H_2_O	High	7	4	Yes	-	Yes	Yes	-	Yes	NA	-	NA
		Low	7	7	Yes	Yes	Yes	Yes	Yes	Yes	NA	Yes	NA
	Human serum	High	7	7	Yes	Yes	Yes	Yes	Yes	Yes	NA	Yes	NA
		Low	7	4	Yes	-	Yes	Yes	-	Yes	NA	-	NA
	Murine serum	High	7	5	Yes	-	Yes	Yes	-	Yes	NA	Yes	NA
		Low	7	3	Yes	-	Yes	-	-	Yes	NA	-	NA
Center 5	MeOH:H_2_O	High	9	9	Yes	Yes	Yes	Yes	Yes	Yes	Yes	Yes	Yes
		Low	9	9	Yes	Yes	Yes	Yes	Yes	Yes	Yes	Yes	Yes
	Human serum	High	9	9	Yes	Yes	Yes	Yes	Yes	Yes	Yes	Yes	Yes
		Low	9	9	Yes	Yes	Yes	Yes	Yes	Yes	Yes	Yes	Yes
	Murine serum	High	9	9	Yes	Yes	Yes	Yes	Yes	Yes	Yes	Yes	Yes
		Low	9	8	Yes	Yes	Yes	-	Yes	Yes	Yes	Yes	Yes
Center 6	MeOH:H_2_O	High	9	9	Yes	Yes	Yes	Yes	Yes	Yes	Yes	Yes	Yes
		Low	9	8	Yes	Yes	Yes	Yes	Yes	-	Yes	Yes	Yes
	Human serum	High	9	9	Yes	Yes	Yes	Yes	Yes	Yes	Yes	Yes	Yes
		Low	9	7	Yes	Yes	Yes	Yes	-	-	Yes	Yes	Yes
	Murine serum	High	9	8	Yes	Yes	Yes	Yes	-	Yes	Yes	Yes	Yes
		Low	9	7	Yes	Yes	Yes	-	Yes	-	Yes	Yes	Yes

**Table 3:** Bile acid analyses within accuracy limits based on relative recovery. NA = not applicable, those BAs are not covered by the specific center.

Center	Matrix	Conc. Level	# BAs in Method	# BAs in Rel. Recovery Limits	Bile Acid in Relative Recovery Limits								
					CA	CDCA	GCA	TCA	DCA	LCA	α-MCA	β-MCA	ω-MCA
Center 1	MeOH:H_2_O	High	9	2	-	-	Yes	Yes	-	-	-	-	-
		Low	9	2	-	-	Yes	Yes	-	-	-	-	-
	Human serum	High	9	0	-	-	-	-	-	-	-	-	-
		Low	9	1	-	-	-	Yes	-	-	-	-	-
	Murine serum	High	9	1	-	-	-	Yes	-	-	-	-	-
		Low	9	2	-	-	Yes	Yes	-	-	-	-	-
Center 2	MeOH:H_2_O	High	9	5	Yes	-	Yes	Yes	Yes	-	-	Yes	-
		Low	9	2	-	Yes	Yes	-	-	-	-	-	-
	Human serum	High	9	1	-	Yes	-	-	-	-	-	-	-
		Low	9	5	Yes	-	Yes	Yes	Yes	-	-	Yes	-
	Murine serum	High	9	4	Yes	-	Yes	Yes	Yes	-	-	-	-
		Low	9	3	Yes	-	-	Yes	Yes	-	-	-	-
Center 3	MeOH:H_2_O	High	7	5	Yes	-	Yes	Yes	Yes	NA	-	Yes	NA
		Low	7	5	Yes	-	Yes	Yes	Yes	NA	Yes	-	NA
	Human serum	High	7	5	Yes	-	Yes	Yes	Yes	NA	-	Yes	NA
		Low	7	5	Yes	-	Yes	Yes	Yes	NA	Yes	-	NA
	Murine serum	High	7	6	Yes	-	Yes	Yes	Yes	NA	Yes	Yes	NA
		Low	7	6	Yes	-	Yes	Yes	Yes	NA	Yes	Yes	NA
Center 4	MeOH:H_2_O	High	7	2	-	-	Yes	Yes	-	-	NA	-	NA
		Low	7	3	Yes	-	Yes	Yes	-	-	NA	-	NA
	Human serum	High	7	4	Yes	-	Yes	Yes	-	Yes	NA	-	NA
		Low	7	3	Yes	-	Yes	Yes	-	-	NA	-	NA
	Murine serum	High	7	3	Yes	-	Yes	Yes	-	-	NA	-	NA
		Low	7	2	Yes	-	Yes	-	-	-	NA	-	NA
Center 5	MeOH:H_2_O	High	9	4	-	Yes	-	Yes	-	-	Yes	Yes	-
		Low	9	6	Yes	-	Yes	Yes	-	-	Yes	Yes	Yes
	Human serum	High	9	5	-	Yes	-	Yes	Yes	-	Yes	Yes	-
		Low	9	5	Yes	-	Yes	Yes	-	-	Yes	Yes	-
	Murine serum	High	9	3	-	-	-	Yes	Yes	-	-	Yes	-
		Low	9	4	Yes	-	Yes	-	-	-	Yes	Yes	-
Center 6	MeOH:H_2_O	High	9	6	Yes	Yes	Yes	Yes	Yes	-	Yes	-	-
		Low	9	7	Yes	Yes	Yes	Yes	Yes	-	Yes	-	Yes
	Human serum	High	9	7	Yes	Yes	Yes	Yes	Yes	-	Yes	-	Yes
		Low	9	7	Yes	Yes	Yes	Yes	Yes	-	Yes	-	Yes
	Murine serum	High	9	6	Yes	Yes	Yes	Yes	Yes	-	Yes	-	-
		Low	9	6	Yes	Yes	Yes	Yes	Yes	-	Yes	-	-

**Table 4:** Summary of the methodology used by the individual centers.

Center	Column Type	Mass Spectrometer	Flow Rate (mL/min)	Analysis Time (min)	Total Turn-Around Time (min)	Sample Volume Required for BA Extraction (µL)	Sample Volume Injected (µL)	Software	Reference
Center 1	ACQUITY UPLC BEH Shield RP18 column (particle size: 1.7 µm, dimensions: 100 × 2.1 mm)	Xevo TQ-S (Waters)	0.7	13	19	25	5	Target Lynx v4.1 (Waters)	García-Cañaveras et al. [23]
Center 2	Kinetex C18 reversed phase column (particle size: 2.6 μm, dimensions: 100 × 3.0 mm)	Q Exactive hybrid quadrupole-Orbitrap (Thermo Fisher Scientific)	0.5	23	25	10	10	Xcalibur 2.3 (Thermo Fisher Scientific)	Amplatz et al. [24]
Center 3	Kinetex C18 reversed phase column (particle size: 1.7 μm, dimensions: 100 × 2.1 mm) and a SecurityGuard ULTRA Cartridges UHPLC C18 2.1 mm column	QTrap 5500 (Sciex)	0.4	23	25	30	1	Multiquant 3.0.3 (Sciex)	Reiter et al. [25]
Center 4	ACQUITY UPLC BEH Shield RP18 column (particle size: 1.7 µm, dimensions: 50 × 2.1 mm)	Xevo TQ-S (Waters)	0.4	18.6	20	50	5	Target Lynx v4.1 (Waters)	Tagliacozzi et al. [26]
Center 5	Kinetex C18 reversed phase column (particle size: 1.7 μm, dimensions: 100 × 2.1 mm)	QTrap 5500 (Sciex)	0.4	18.6	20	50	5	Multiquant 3.0.3 (Sciex)	García-Cañaveras et al. [23]
Center 6	ACQUITY UPLC System (UHPLC Column from Biocrates P.-No 91220052120868)	QTrap 5500 (Sciex)	0.5	3.5	5	10	5	Analyst 1.7.1 (Sciex)	Phamet al. [22]

**Table 5:** LC-gradient conditions for center 1.

	Time (min)	Flow Rate (mL/min)	Mobile Phase A (%)
Analysis	0	0.7	85
	9	0.7	60
	10	0.7	20
	13	0.7	0
Column Regeneration	16	0.7	0
	16.1	0.7	85
	19	0.7	85

**Table 6:** LC-gradient for center 2.

	Time (min)	Flow Rate (mL/min)	Mobile Phase A (%)
Analysis	0	0.4	75
	2	0.4	75
	3.5	0.4	73
	5.5	0.4	65
	10	0.4	65
	11	0.4	57
	12	0.4	57
	14	0.4	42
	17	0.4	42
	17.5	0.4	35
	18	0.4	20
	19	0.4	0
	20	0.4	0
	23	0.4	75
Column Regeneration	25	0.4	75

**Table 7:** LC-gradient conditions for center 3.

	Time (min)	Flow Rate (mL/min)	Mobile Phase A (%)
Analysis	0	0.4	75
	2	0.4	75
	3.5	0.4	73
	5.5	0.4	65
	10	0.4	65
	11	0.4	57
	12	0.4	57
	14	0.4	42
	17	0.4	42
	17.5	0.4	35
	18	0.4	20
	19	0.4	0
	20	0.4	0
	23	0.4	75
Column Regeneration	25	0.4	75

**Table 8:** LC-gradient conditions center 4.

	Time (min)	Flow Rate (mL/min)	Mobile Phase A (%)
Analysis	0	0.4	80
	1	0.4	81
	5	0.4	65
	14.5	0.4	5
	14.6	0.4	0
	18.5	0.4	0
	18.6	0.4	80
Column Regeneration	20	0.4	80

**Table 9:** LC-gradient conditions for center 5.

	Time (min)	Flow Rate (mL/min)	Mobile Phase A (%)
Analysis	0	0.4	80
	1	0.4	80
	5	0.4	65
	14.5	0.4	5
	14.6	0.4	0
	18.5	0.4	0
	18.6	0.4	80
Column Regeneration	20	0.4	80

**Table 10:** LC-gradient conditions for center 6.

	Time (min)	Flow Rate (mL/min)	Mobile Phase A (%)
Analysis	0	0.5	65
	0.25	0.5	65
	0.35	0.5	60
	1.9	0.5	55
	2.1	0.8	45
	3.3	1.0	35
	3.5	1.0	0
Column Regeneration	4.0	1.0	0
	4.1	0.9	65
	5	0.5	65

## Data Availability

Data is included in the Supplemental Table S1.

## Supplementary Materials

The following supporting information can be downloaded at: https://www.mdpi.com/article/10.3390/metabo12070583/s1, Supplemental Table S1.
