# Supplementary material for: Ring Trial on Quantitative Assessment of Bile Acids Reveals a Method- and Analyte-Specific Accuracy and Reproducibility
Source: Metabolites. 2022 Jun 23;12(7):583. doi: 10.3390/metabo12070583 (PMC9319092; doi:10.3390/metabo12070583)
Supplement: Supplementary file 1 [file metabolites-12-00583-s001.zip › metabolites-1755381-supplementary.pdf]

Supplemental material

Supplemental Table S1: Mean bile acid concentrations determined at each center

Center	Matrix	Concentration level	Bile acid	Mean concentration (nmol/L)	Standard deviation
Center_1	MeOH:H2O	Low concentration	CA	1542.7	200.3
Center_1	MeOH:H2O	Low concentration	CDCA	756.1	94.0
Center_1	MeOH:H2O	Low concentration	GCA	328.6	24.1
Center_1	MeOH:H2O	Low concentration	TCA	217.6	10.7
Center_1	MeOH:H2O	Low concentration	DCA	340.9	40.9
Center_1	MeOH:H2O	Low concentration	LCA	61.0	6.1
Center_1	MeOH:H2O	Low concentration	α-MCA	457.3	68.8
Center_1	MeOH:H2O	Low concentration	β-MCA	1161.2	225.8
Center_1	MeOH:H2O	Low concentration	ω-MCA	264.6	46.7
Center_1	MeOH:H2O	High concentration	CA	10589.1	1028.7
Center_1	MeOH:H2O	High concentration	CDCA	6825.4	429.3
Center_1	MeOH:H2O	High concentration	GCA	36723.3	3468.6
Center_1	MeOH:H2O	High concentration	TCA	20048.3	1905.8
Center_1	MeOH:H2O	High concentration	DCA	2932.2	215.9
Center_1	MeOH:H2O	High concentration	LCA	603.1	37.7
Center_1	MeOH:H2O	High concentration	α-MCA	4178.8	575.4
Center_1	MeOH:H2O	High concentration	β-MCA	10256.4	1309.5
Center_1	MeOH:H2O	High concentration	ω-MCA	2468.2	335.1
Center_1	Human serum	Low concentration	CA	1533.5	157.1
Center_1	Human serum	Low concentration	CDCA	427.5	60.5
Center_1	Human serum	Low concentration	GCA	152.8	17.5
Center_1	Human serum	Low concentration	TCA	213.5	31.4
Center_1	Human serum	Low concentration	DCA	186.7	23.6
Center_1	Human serum	Low concentration	LCA	43.6	3.5
Center_1	Human serum	Low concentration	α-MCA	516.5	78.5
Center_1	Human serum	Low concentration	β-MCA	1357.9	207.3
Center_1	Human serum	Low concentration	ω-MCA	314.0	58.8
Center_1	Human serum	High concentration	CA	12526.1	1834.8
Center_1	Human serum	High concentration	CDCA	5215.9	554.3
Center_1	Human serum	High concentration	GCA	20167.5	2328.9
Center_1	Human serum	High concentration	TCA	10539.8	1311.5
Center_1	Human serum	High concentration	DCA	1989.1	169.8
Center_1	Human serum	High concentration	LCA	629.2	58.5
Center_1	Human serum	High concentration	α-MCA	4378.3	729.4
Center_1	Human serum	High concentration	β-MCA	11224.4	2076.3
Center_1	Human serum	High concentration	ω-MCA	2914.7	507.7

Center_1	Mouse serum	Low concentration	CA	1363.1	357.5
Center_1	Mouse serum	Low concentration	CDCA	693.4	86.8
Center_1	Mouse serum	Low concentration	GCA	465.9	55.3
Center_1	Mouse serum	Low concentration	TCA	189.4	542.3
Center_1	Mouse serum	Low concentration	DCA	292.5	38.7
Center_1	Mouse serum	Low concentration	LCA	49.8	5.7
Center_1	Mouse serum	Low concentration	α-MCA	545.7	266.5
Center_1	Mouse serum	Low concentration	β-MCA	972.0	510.2
Center_1	Mouse serum	Low concentration	ω-MCA	239.1	105.6
Center_1	Mouse serum	High concentration	CA	10374.5	1831.5
Center_1	Mouse serum	High concentration	CDCA	6437.7	396.8
Center_1	Mouse serum	High concentration	GCA	53212.3	10623.3
Center_1	Mouse serum	High concentration	TCA	26911.0	4348.4
Center_1	Mouse serum	High concentration	DCA	2787.4	218.6
Center_1	Mouse serum	High concentration	LCA	566.1	95.1
Center_1	Mouse serum	High concentration	α-MCA	3914.7	669.5
Center_1	Mouse serum	High concentration	β-MCA	9564.6	1963.9
Center_1	Mouse serum	High concentration	ω-MCA	2510.5	506.4
Center_2	MeOH:H2O	Low concentration	CA	1686.2	614.6
Center_2	MeOH:H2O	Low concentration	CDCA	1077.7	425.6
Center_2	MeOH:H2O	Low concentration	GCA	298.1	153.4
Center_2	MeOH:H2O	Low concentration	TCA	142.3	66.1
Center_2	MeOH:H2O	Low concentration	DCA	305.2	114.7
Center_2	MeOH:H2O	Low concentration	LCA	51.4	18.0
Center_2	MeOH:H2O	Low concentration	α-MCA	0.0	0.0
Center_2	MeOH:H2O	Low concentration	β-MCA	162.1	63.7
Center_2	MeOH:H2O	Low concentration	ω-MCA	0.0	0.0
Center_2	MeOH:H2O	High concentration	CA	23342.7	2294.7
Center_2	MeOH:H2O	High concentration	CDCA	17631.4	1847.3
Center_2	MeOH:H2O	High concentration	GCA	35781.7	3446.5
Center_2	MeOH:H2O	High concentration	TCA	22680.4	2439.7
Center_2	MeOH:H2O	High concentration	DCA	4161.9	393.5
Center_2	MeOH:H2O	High concentration	LCA	794.3	68.7
Center_2	MeOH:H2O	High concentration	α-MCA	671.3	73.6
Center_2	MeOH:H2O	High concentration	β-MCA	2310.9	268.9
Center_2	MeOH:H2O	High concentration	ω-MCA	700.0	83.9
Center_2	Human serum	Low concentration	CA	2880.1	118.6
Center_2	Human serum	Low concentration	CDCA	2727.1	121.6
Center_2	Human serum	Low concentration	GCA	454.2	28.2
Center_2	Human serum	Low concentration	TCA	177.1	23.4
Center_2	Human serum	Low concentration	DCA	515.5	43.0
Center_2	Human serum	Low concentration	LCA	46.4	3.5

Center_2	Human serum	Low concentration	α-MCA	29.8	4.7
Center_2	Human serum	Low concentration	β-MCA	264.7	18.5
Center_2	Human serum	Low concentration	ω-MCA	29.3	4.4
Center_2	Human serum	High concentration	CA	14153.6	720.3
Center_2	Human serum	High concentration	CDCA	10737.0	433.5
Center_2	Human serum	High concentration	GCA	21388.2	1195.9
Center_2	Human serum	High concentration	TCA	12577.3	2002.0
Center_2	Human serum	High concentration	DCA	2368.9	101.6
Center_2	Human serum	High concentration	LCA	510.2	22.6
Center_2	Human serum	High concentration	α-MCA	402.1	16.7
Center_2	Human serum	High concentration	β-MCA	1482.8	52.5
Center_2	Human serum	High concentration	ω-MCA	411.3	75.7
Center_2	Mouse serum	Low concentration	CA	3024.0	397.4
Center_2	Mouse serum	Low concentration	CDCA	2101.8	222.5
Center_2	Mouse serum	Low concentration	GCA	535.9	123.2
Center_2	Mouse serum	Low concentration	TCA	269.9	106.4
Center_2	Mouse serum	Low concentration	DCA	549.5	72.1
Center_2	Mouse serum	Low concentration	LCA	84.7	13.2
Center_2	Mouse serum	Low concentration	α-MCA	90.6	18.1
Center_2	Mouse serum	Low concentration	β-MCA	331.2	118.0
Center_2	Mouse serum	Low concentration	ω-MCA	87.6	30.3
Center_2	Mouse serum	High concentration	CA	30914.7	2542.1
Center_2	Mouse serum	High concentration	CDCA	23992.8	1818.0
Center_2	Mouse serum	High concentration	GCA	46946.5	3690.8
Center_2	Mouse serum	High concentration	TCA	27030.0	2291.2
Center_2	Mouse serum	High concentration	DCA	5604.8	480.6
Center_2	Mouse serum	High concentration	LCA	1084.1	94.7
Center_2	Mouse serum	High concentration	α-MCA	947.4	77.0
Center_2	Mouse serum	High concentration	β-MCA	3203.9	251.7
Center_2	Mouse serum	High concentration	ω-MCA	944.7	98.2
Center_3	MeOH:H2O	Low concentration	CA	2383.4	157.0
Center_3	MeOH:H2O	Low concentration	CDCA	974.8	119.0
Center_3	MeOH:H2O	Low concentration	GCA	404.7	74.4
Center_3	MeOH:H2O	Low concentration	TCA	234.2	35.3
Center_3	MeOH:H2O	Low concentration	DCA	479.8	75.6
Center_3	MeOH:H2O	Low concentration	α-MCA	160.5	0.0
Center_3	MeOH:H2O	Low concentration	β-MCA	410.8	82.3
Center_3	MeOH:H2O	High concentration	CA	21865.1	1655.0
Center_3	MeOH:H2O	High concentration	CDCA	9584.4	725.5
Center_3	MeOH:H2O	High concentration	GCA	34093.5	2155.7
Center_3	MeOH:H2O	High concentration	TCA	21283.0	1391.1
Center_3	MeOH:H2O	High concentration	DCA	4386.7	582.0

Center_3	MeOH:H2O	High concentration	α-MCA	1036.6	153.2
Center_3	MeOH:H2O	High concentration	β-MCA	2898.0	196.8
Center_3	Human serum	Low concentration	CA	2113.7	202.3
Center_3	Human serum	Low concentration	CDCA	902.0	203.0
Center_3	Human serum	Low concentration	GCA	342.5	52.2
Center_3	Human serum	Low concentration	TCA	249.4	41.6
Center_3	Human serum	Low concentration	DCA	410.5	128.5
Center_3	Human serum	Low concentration	α-MCA	150.6	0.0
Center_3	Human serum	Low concentration	β-MCA	368.4	64.7
Center_3	Human serum	High concentration	CA	22658.4	1945.3
Center_3	Human serum	High concentration	CDCA	9901.0	1004.2
Center_3	Human serum	High concentration	GCA	33136.5	4229.1
Center_3	Human serum	High concentration	TCA	21250.1	1652.0
Center_3	Human serum	High concentration	DCA	4451.5	435.2
Center_3	Human serum	High concentration	α-MCA	986.1	84.8
Center_3	Human serum	High concentration	β-MCA	3003.2	523.8
Center_3	Mouse serum	Low concentration	CA	2231.7	322.1
Center_3	Mouse serum	Low concentration	CDCA	818.4	110.2
Center_3	Mouse serum	Low concentration	GCA	382.0	73.6
Center_3	Mouse serum	Low concentration	TCA	257.6	231.1
Center_3	Mouse serum	Low concentration	DCA	482.3	84.0
Center_3	Mouse serum	Low concentration	α-MCA	184.0	30.7
Center_3	Mouse serum	Low concentration	β-MCA	233.6	87.4
Center_3	Mouse serum	High concentration	CA	21489.6	3507.4
Center_3	Mouse serum	High concentration	CDCA	8684.3	1110.1
Center_3	Mouse serum	High concentration	GCA	34520.8	4024.7
Center_3	Mouse serum	High concentration	TCA	18297.1	2064.6
Center_3	Mouse serum	High concentration	DCA	3956.0	361.7
Center_3	Mouse serum	High concentration	α-MCA	1079.1	144.8
Center_3	Mouse serum	High concentration	β-MCA	2496.2	455.7
Center_4	MeOH:H2O	Low concentration	CA	2125.2	145.2
Center_4	MeOH:H2O	Low concentration	CDCA	1002.3	93.1
Center_4	MeOH:H2O	Low concentration	GCA	324.0	27.4
Center_4	MeOH:H2O	Low concentration	TCA	211.9	20.6
Center_4	MeOH:H2O	Low concentration	DCA	315.0	54.4
Center_4	MeOH:H2O	Low concentration	LCA	79.1	10.3
Center_4	MeOH:H2O	Low concentration	β-MCA	3.0	0.7
Center_4	MeOH:H2O	High concentration	CA	19877.1	2871.2
Center_4	MeOH:H2O	High concentration	CDCA	7832.0	4389.9
Center_4	MeOH:H2O	High concentration	GCA	28865.2	4657.4
Center_4	MeOH:H2O	High concentration	TCA	18725.9	3021.9
Center_4	MeOH:H2O	High concentration	DCA	2627.6	1596.4

Center_4	MeOH:H2O	High concentration	LCA	881.6	86.9
Center_4	MeOH:H2O	High concentration	β-MCA	24.7	15.0
Center_4	Human serum	Low concentration	CA	2286.8	60.3
Center_4	Human serum	Low concentration	CDCA	840.7	379.6
Center_4	Human serum	Low concentration	GCA	353.7	20.7
Center_4	Human serum	Low concentration	TCA	235.8	6.5
Center_4	Human serum	Low concentration	DCA	53.8	255.8
Center_4	Human serum	Low concentration	LCA	77.3	6.9
Center_4	Human serum	Low concentration	β-MCA	3.4	1.4
Center_4	Human serum	High concentration	CA	22701.3	447.6
Center_4	Human serum	High concentration	CDCA	8548.3	1330.2
Center_4	Human serum	High concentration	GCA	34475.3	1767.4
Center_4	Human serum	High concentration	TCA	21495.3	1025.9
Center_4	Human serum	High concentration	DCA	2644.2	640.4
Center_4	Human serum	High concentration	LCA	1117.5	99.1
Center_4	Human serum	High concentration	β-MCA	33.8	5.5
Center_4	Mouse serum	Low concentration	CA	2183.9	189.8
Center_4	Mouse serum	Low concentration	CDCA	763.8	284.2
Center_4	Mouse serum	Low concentration	GCA	360.8	22.5
Center_4	Mouse serum	Low concentration	TCA	36.9	41.3
Center_4	Mouse serum	Low concentration	DCA	344.1	236.8
Center_4	Mouse serum	Low concentration	LCA	88.1	18.2
Center_4	Mouse serum	Low concentration	β-MCA	607.5	1099.2
Center_4	Mouse serum	High concentration	CA	22554.8	1083.1
Center_4	Mouse serum	High concentration	CDCA	6187.9	2437.5
Center_4	Mouse serum	High concentration	GCA	32374.4	1571.0
Center_4	Mouse serum	High concentration	TCA	20150.9	1307.0
Center_4	Mouse serum	High concentration	DCA	2417.3	1146.7
Center_4	Mouse serum	High concentration	LCA	1010.9	128.8
Center_4	Mouse serum	High concentration	β-MCA	-1036.3	48.8
Center_5	MeOH:H2O	Low concentration	CA	2979.6	164.1
Center_5	MeOH:H2O	Low concentration	CDCA	1042.8	20.0
Center_5	MeOH:H2O	Low concentration	GCA	346.2	7.4
Center_5	MeOH:H2O	Low concentration	TCA	266.7	5.4
Center_5	MeOH:H2O	Low concentration	DCA	311.5	7.4
Center_5	MeOH:H2O	Low concentration	LCA	33.3	1.8
Center_5	MeOH:H2O	Low concentration	α-MCA	120.8	5.5
Center_5	MeOH:H2O	Low concentration	β-MCA	231.9	10.1
Center_5	MeOH:H2O	Low concentration	ω-MCA	107.4	4.4
Center_5	MeOH:H2O	High concentration	CA	16509.2	506.4
Center_5	MeOH:H2O	High concentration	CDCA	10530.4	347.3
Center_5	MeOH:H2O	High concentration	GCA	26586.1	354.3

Center_5	MeOH:H2O	High concentration	TCA	22150.8	503.8
Center_5	MeOH:H2O	High concentration	DCA	3223.1	294.0
Center_5	MeOH:H2O	High concentration	LCA	417.5	16.4
Center_5	MeOH:H2O	High concentration	α-MCA	1121.8	32.9
Center_5	MeOH:H2O	High concentration	β-MCA	1997.7	65.1
Center_5	MeOH:H2O	High concentration	ω-MCA	1024.8	35.3
Center_5	Human serum	Low concentration	CA	2584.8	61.5
Center_5	Human serum	Low concentration	CDCA	988.7	12.7
Center_5	Human serum	Low concentration	GCA	300.4	4.7
Center_5	Human serum	Low concentration	TCA	235.3	4.4
Center_5	Human serum	Low concentration	DCA	315.9	15.6
Center_5	Human serum	Low concentration	LCA	34.1	1.5
Center_5	Human serum	Low concentration	α-MCA	108.7	1.6
Center_5	Human serum	Low concentration	β-MCA	203.7	2.2
Center_5	Human serum	Low concentration	ω-MCA	96.9	1.5
Center_5	Human serum	High concentration	CA	15718.3	445.5
Center_5	Human serum	High concentration	CDCA	11100.9	318.5
Center_5	Human serum	High concentration	GCA	25772.7	428.5
Center_5	Human serum	High concentration	TCA	22743.7	724.1
Center_5	Human serum	High concentration	DCA	4127.6	280.6
Center_5	Human serum	High concentration	LCA	456.3	23.7
Center_5	Human serum	High concentration	α-MCA	1130.1	19.3
Center_5	Human serum	High concentration	β-MCA	1998.6	38.7
Center_5	Human serum	High concentration	ω-MCA	1038.4	24.9
Center_5	Mouse serum	Low concentration	CA	2935.6	217.6
Center_5	Mouse serum	Low concentration	CDCA	1016.0	27.1
Center_5	Mouse serum	Low concentration	GCA	309.9	6.3
Center_5	Mouse serum	Low concentration	TCA	107.8	39.1
Center_5	Mouse serum	Low concentration	DCA	312.2	9.3
Center_5	Mouse serum	Low concentration	LCA	34.3	1.4
Center_5	Mouse serum	Low concentration	α-MCA	108.8	6.3
Center_5	Mouse serum	Low concentration	β-MCA	200.9	24.5
Center_5	Mouse serum	Low concentration	ω-MCA	75.0	8.3
Center_5	Mouse serum	High concentration	CA	15982.8	404.7
Center_5	Mouse serum	High concentration	CDCA	10430.5	330.5
Center_5	Mouse serum	High concentration	GCA	25167.1	456.3
Center_5	Mouse serum	High concentration	TCA	20516.0	804.4
Center_5	Mouse serum	High concentration	DCA	3680.8	339.3
Center_5	Mouse serum	High concentration	LCA	405.2	9.7
Center_5	Mouse serum	High concentration	α-MCA	1045.2	26.5
Center_5	Mouse serum	High concentration	β-MCA	1871.2	45.1
Center_5	Mouse serum	High concentration	ω-MCA	952.6	25.6

Center_6	MeOH:H2O	Low concentration	CA	2746.7	110.9
Center_6	MeOH:H2O	Low concentration	CDCA	1458.3	103.8
Center_6	MeOH:H2O	Low concentration	GCA	432.2	21.8
Center_6	MeOH:H2O	Low concentration	TCA	225.3	15.3
Center_6	MeOH:H2O	Low concentration	DCA	533.2	103.6
Center_6	MeOH:H2O	Low concentration	LCA	91.0	39.8
Center_6	MeOH:H2O	Low concentration	α-MCA	142.0	10.1
Center_6	MeOH:H2O	Low concentration	β-MCA	492.7	18.7
Center_6	MeOH:H2O	Low concentration	ω-MCA	120.5	10.7
Center_6	MeOH:H2O	High concentration	CA	25766.7	1714.3
Center_6	MeOH:H2O	High concentration	CDCA	14400.0	853.2
Center_6	MeOH:H2O	High concentration	GCA	37566.7	3072.2
Center_6	MeOH:H2O	High concentration	TCA	20300.0	1473.8
Center_6	MeOH:H2O	High concentration	DCA	5020.0	521.3
Center_6	MeOH:H2O	High concentration	LCA	961.5	97.6
Center_6	MeOH:H2O	High concentration	α-MCA	1186.7	96.7
Center_6	MeOH:H2O	High concentration	β-MCA	3750.0	225.9
Center_6	MeOH:H2O	High concentration	ω-MCA	1041.5	55.3
Center_6	Human serum	Low concentration	CA	2747.0	159.2
Center_6	Human serum	Low concentration	CDCA	1193.5	214.4
Center_6	Human serum	Low concentration	GCA	435.9	33.2
Center_6	Human serum	Low concentration	TCA	231.1	19.0
Center_6	Human serum	Low concentration	DCA	612.8	201.0
Center_6	Human serum	Low concentration	LCA	65.0	26.2
Center_6	Human serum	Low concentration	α-MCA	135.8	14.6
Center_6	Human serum	Low concentration	β-MCA	471.2	24.6
Center_6	Human serum	Low concentration	ω-MCA	117.0	6.5
Center_6	Human serum	High concentration	CA	27615.3	1506.5
Center_6	Human serum	High concentration	CDCA	14216.8	1090.7
Center_6	Human serum	High concentration	GCA	49339.4	7324.7
Center_6	Human serum	High concentration	TCA	24840.8	3653.7
Center_6	Human serum	High concentration	DCA	6291.2	171.4
Center_6	Human serum	High concentration	LCA	911.3	120.0
Center_6	Human serum	High concentration	α-MCA	1306.7	88.0
Center_6	Human serum	High concentration	β-MCA	4176.7	306.6
Center_6	Human serum	High concentration	ω-MCA	1121.7	53.1
Center_6	Mouse serum	Low concentration	CA	2693.3	304.4
Center_6	Mouse serum	Low concentration	CDCA	1132.2	93.7
Center_6	Mouse serum	Low concentration	GCA	448.3	28.3
Center_6	Mouse serum	Low concentration	TCA	184.0	57.6
Center_6	Mouse serum	Low concentration	DCA	597.3	33.6
Center_6	Mouse serum	Low concentration	LCA	47.7	0.0

Center_6	Mouse serum	Low concentration	α-MCA	132.8	24.1
Center_6	Mouse serum	Low concentration	β-MCA	398.3	113.7
Center_6	Mouse serum	Low concentration	ω-MCA	81.3	23.0
Center_6	Mouse serum	High concentration	CA	25246.7	1028.9
Center_6	Mouse serum	High concentration	CDCA	13833.8	2358.0
Center_6	Mouse serum	High concentration	GCA	35616.7	3028.8
Center_6	Mouse serum	High concentration	TCA	20004.0	1856.9
Center_6	Mouse serum	High concentration	DCA	5836.0	2330.3
Center_6	Mouse serum	High concentration	LCA	868.8	159.0
Center_6	Mouse serum	High concentration	α-MCA	1207.5	41.8
Center_6	Mouse serum	High concentration	β-MCA	3471.7	197.6
Center_6	Mouse serum	High concentration	ω-MCA	921.0	78.3
